# Association of Visceral Fat Mass Index with Diabetes and Vascular Complications: NHANES 2011–2018 Cross-Sectional Study

**DOI:** 10.34133/hds.0441

**Published:** 2026-03-10

**Authors:** Yao Wang, Xingyu He, Guangya Xu, Jiahao Zhang, Na Li, Xiangming Ning, Jun Ma, Hongling Li, Jingxian Yang, Hao Liu, Jiayi Hou, Wenjun Wei, Junming Ren, Jingjing Huang, Xian Shao, Zheng Shi

**Affiliations:** ^1^ Clinical Medical College & Affiliated Hospital of Chengdu University, Chengdu University, Chengdu, Sichuan 610081, China.; ^2^ College of Pharmacy, Chengdu University, Chengdu, Sichuan 610081, China.; ^3^ College of Basic Medical Sciences, Chengdu University, Chengdu, Sichuan 610081, China.; ^4^ Cardiac Structure and Function Laboratory, West China Hospital, Sichuan University, Chengdu 610041, China.; ^5^ Department of Clinical Pharmacy, School of Pharmacy, Zunyi Medical University, Zunyi, Guizhou 563006, China.; ^6^ Division of Nephrology, National Clinical Research Center for Kidney Disease, State Key Laboratory of Organ Failure Research, Nanfang Hospital, Southern Medical University, Guangzhou 510000, China.

## Abstract

**Background:** Visceral fat mass index (vFMI), a key visceral obesity marker, is linked to insulin resistance and cardiovascular impairment. This study examined the relationship between vFMI, people living with diabetes, and its vascular complications. **Methods:** Data from NHANES (2011–2018) were used, including participants aged 18 years and older. Multivariable logistic regression models examined associations between vFMI and diabetes, diabetic nephropathy (DN), and cardiovascular disease (CVD). **Result:** A total of 4,847 participants were included, comprising 2,469 (50.9%) males and 2,378 (49.1%) females, with a mean age of 40 years. The prevalence of diabetes, DN, and CVD was 11.6% (567), 2.8% (136), and 4.3% (208), respectively. Participants in the highest vFMI quartile had a markedly higher risk of diabetes [odds ratio (OR): 5.85, 95% confidence interval (CI): 3.25 to 10.55], DN (OR: 2.84, 95% CI: 0.89 to 9.08), and CVD (OR: 2.55, 95% CI: 1.10 to 5.93) versus the lowest quartile. Nonlinear regression identified critical thresholds for log-transformed vFMI, beyond which risks sharply increased: 0.252 for diabetes and 0.241 for DN. Subgroup analysis showed stronger associations in younger participants (≤40 years), those with lower triglyceride levels, and those with higher β-cell function. **Conclusion:** Higher vFMI correlates with greater prevalence of diabetes and related complications, indicating that visceral fat plays a critical role in their development.

## Introduction

Diabetes is a chronic endocrine disorder that can lead to vascular complications such as diabetic nephropathy (DN) and cardiovascular disease (CVD) through a multifactorial pathway, posing a significant challenge to global public health for people living with diabetes [1]. According to the International Diabetes Federation (IDF), by 2021, the number of people living with diabetes globally had exceeded 530 million, resulting in up to 6.7 million deaths, with an estimated 44.7% of cases remaining undiagnosed [2]. The global number of diabetes cases has increased nearly 4-fold over the past 30 years [3]. In recent years, researchers have increasingly focused on the close association between diabetes and visceral obesity, particularly visceral adipose tissue (VAT). This tissue has been shown to be closely associated with insulin resistance, atherosclerosis, and immune activation [4,5]. Excess accumulation of ectopic fat has been associated with increased risks of diabetes, microvascular kidney damage, and CVD [6–8]. Therefore, accurately assessing visceral obesity is crucial for the diagnosis and treatment of diabetes.

Currently, the commonly used clinical indicators for VAT assessment include body mass index (BMI), waist circumference (WC), and visceral adiposity index (VAI) [7,9,10]. The emerging visceral fat mass index (vFMI) provides a more direct measure of VAT by quantifying the distribution of visceral fat per unit of height squared through imaging techniques, thereby reducing variability in results caused by differences in body shape [11,12]. Computed tomography (CT) and magnetic resonance imaging (MRI) are considered the gold standards for vFMI assessment, but dual-energy x-ray absorptiometry (DXA) has been shown to provide comparable accuracy with lower radiation exposure and cost. Moreover, DXA can simultaneously assess vFMI and bone mineral density, enabling timely monitoring of osteoporosis and visceral obesity risks in patients with endocrine and metabolic disorders, such as parathyroid disease and obesity [13,14]. This enhances the efficiency and cost-effectiveness of clinical evaluations, offering a promising approach for vFMI assessment [11,15,16]. Previous study explored the relationship between vFMI and glycemic control and demonstrated that vFMI was a more accurate indicator of glycemic status than BMI and WC, but lacked investigation on the relationship between vFMI and diabetic vascular complications [17]. Another study found vFMI measured by CT to be a risk factor for diabetes, but CT imaging is more expensive and emits more radiation than DXA [18]. Currently, there is a lack of research on the relationship between DXA-derived vFMI and diabetes.

While the disease burden of early-onset diabetes is rising rapidly and high BMI remains a key independent risk factor, current evidence still lacks systematic assessment and intervention strategies based on body composition, particularly visceral fat [19]. To address these gaps, we designed a cross-sectional study using data from the National Health and Nutrition Examination Survey (NHANES) in the United States. This study aims to investigate the impact of DXA-derived vFMI on diabetes and diabetic vascular complications and to provide evidence for the use of vFMI in the management of diabetes.

## Methods

### Study population and design

This study was a cross-sectional design that used data from the NHANES, which is conducted biennially to assess the health and nutritional status of the U.S. population through a stratified, multistage probability sampling method [20,21]. The NHANES protocol was approved by the Institutional Review Board of the National Center for Health Statistics (NCHS), and all participants provided informed consent prior to their involvement. Detailed datasets and protocols are publicly accessible on the NHANES website (https://www.cdc.gov/nchs/nhanes).

**Fig. 1.**
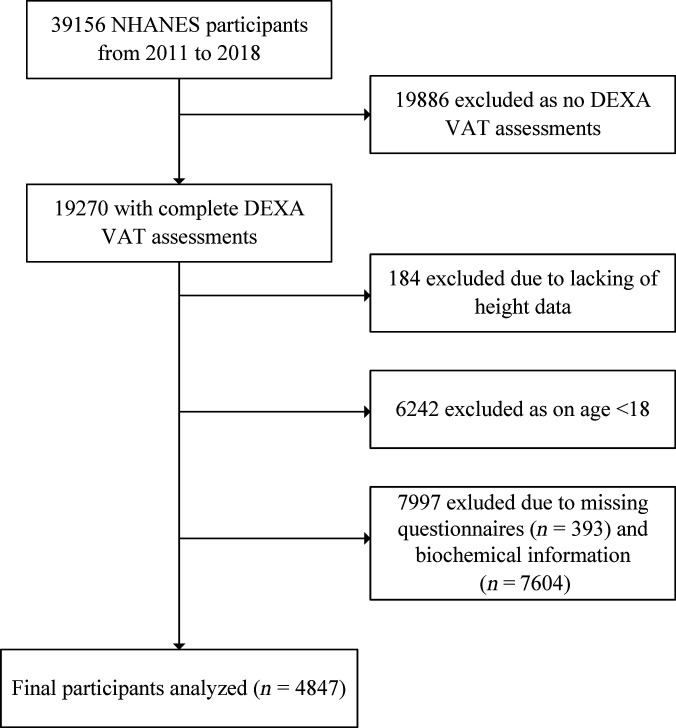
Flow chart of study participants.

Data from 4 NHANES cycles (2011–2018), including 39,156 participants, were initially considered. Several exclusion criteria were applied to derive the final analytic sample. First, participants without DXA-derived VAT assessments (*n* = 19,886) were removed. Among these, 6,275 lacked anthropometric measures (BMI, WC, height, or weight) that are critical for evaluating obesity status; these individuals were therefore excluded from further comparative analysis, leaving 13,611 participants available for descriptive comparison against the final analytic cohort (Table S3). Participants without valid height measurements (*n* = 184) and those younger than 18 years of age (*n* = 6,242) were also excluded. In addition, participants were excluded if they had missing biochemical information required for covariate adjustment, including fasting glucose (*n* = 6,955), fasting insulin (*n* = 7,101), systolic and diastolic blood pressure (SBP and DBP) (*n* = 1,079 each), albumin–creatinine ratio (*n* = 65), triglycerides (*n* = 656), and high-density lipoprotein (HDL) cholesterol (*n* = 603), totaling 7,604 participants. Furthermore, 393 participants were excluded due to missing questionnaire data, including 4 with missing information for diabetes and 38 with missing information for CVD outcomes. After these exclusions, a total of 4,847 participants were included in the final analysis. A detailed flow diagram of participant inclusion and exclusion is provided in Fig. 1.

### Assessment of vFMI

The vFMI is a standardized metric for assessing adiposity, calculated as fat mass (kg) divided by height squared (m^2^). VAT was converted to standard units (kg) and normalized by height squared to derive the vFMI (kg/m^2^) [22,23]. VAT was defined by the Hologic APEX software used in the scan analysis [24]. Measurements of VAT included area, mass, and volume at the L4 to L5 vertebrae level. For subcutaneous adipose tissue, the same vertebral level was used to assess area, mass, and volume. In NHANES, participants aged 18 to 59 years were eligible for VAT assessment, with specific exclusions for pregnant individuals, those who had recently consumed radiographic contrast material, and those exceeding 450 pounds in weight or taller than 6 feet 5 inches. Before the DXA examination, participants were instructed to remove all metal objects from their bodies to ensure measurement accuracy.

### Assessment of diabetes, DN, and CVD prevalence

Diabetes was defined according to the American Diabetes Association (ADA) criteria, which include self-reported physician diagnoses, the use of glucose-lowering medications or insulin, fasting plasma glucose levels ≥ 7.0 mM, or HbA1c ≥ 6.5% in people living with diabetes [25]. DN was identified in participants with diabetes who also had an albumin-to-creatinine ratio (ACR) ≥ 30 mg/g, an estimated glomerular filtration rate (eGFR) < 60 ml/min/1.73 m^2^, or both [26,27]. eGFR was calculated using the Chronic Kidney Disease Epidemiology Collaboration (CKD-EPI) 2021 equation [28].

CVD as determined based on self-reported physician diagnoses. Participants were asked, “Has a doctor or other health professional ever told you that you have congestive heart failure (CHF), coronary heart disease (CHD), angina, myocardial infarction (MI), or stroke?” A positive response to any of these questions was used to classify participants as having CVD [29].

### Assessment of covariates

Information on demographic and health-related factors, such as age, gender, race, education level, poverty income ratio (PIR), smoking status, and alcohol consumption, were collected through household interviews. Smoking status was categorized as current smoker or nonsmoker, and alcohol use was classified based on whether participants consumed at least 12 alcoholic drinks annually.

Clinical examination data included BMI, WC, SBP, and DBP. BMI was calculated as weight (kg) divided by height squared (m^2^). Hypertension was defined as SBP ≥ 130 mmHg or DBP ≥ 90 mmHg, based on the average of 3 measurements, following the 2017 American College of Cardiology/American Heart Association hypertension guidelines [30].

Laboratory data included fasting glucose, insulin concentration, glycated hemoglobin A1c (HbA1c), triglycerides (TGs), HDL cholesterol, and urinary albumin–creatinine ratio (UACR). Insulin resistance and pancreatic β-cell function were assessed using the homeostatic model assessment (HOMA) indices for insulin resistance (HOMA-IR) and β-cell function (HOMA-B) [ 31].

### Covariate selection and model specification

We implemented a hierarchical adjustment strategy to reflect plausible confounding domains. Model 1 was unadjusted. Model 2 adjusted for demographic factors (age, sex, race/ethnicity). Model 3 additionally adjusted for socioeconomic and lifestyle factors (education level, PIR, smoking status, and alcohol consumption). Model 4 further adjusted for clinical risk markers (HDL cholesterol and SBP). Because HDL and SBP may partly lie on the pathway from visceral adiposity to cardiometabolic outcomes, model 4 is interpreted as a conservatively adjusted (partially mediated) effect rather than the total effect. To further validate the exclusion of BMI as a covariate due to its high correlation with vFMI, we performed a sensitivity analysis using model 5, which included BMI as an additional covariate. In addition, we used Pearson correlation coefficient and *R*^2^ to assess the linear relationship between vFMI and BMI.

### Statistical analysis

All statistical analyses were conducted using R software (version 4.3.3) and EmpowerStats (version 4.2). We considered masked variance and used the weighting methodology in all analyses [32]. Categorical variables were expressed as frequencies and percentages (%), while continuous variables were expressed as mean or medians and interquartile ranges (IQRs). The χ^2^ test or Kruskal–Wallis *H* test was used to evaluate differences among the 4 vFMI groups. Statistical significance was determined with a 2-sided *P* value of <0.01.

Multivariable logistic regression models were used to examine the associations between vFMI *z* score and people living with diabetes, DN, and CVD. The vFMI was standardized to facilitate comparisons across participants with differing units and scales. The standardization process was conducted using the *z*-score transformation, which adjusts each value by subtracting the mean ($\bar{x}$) and dividing by the standard deviation (SD) (σ) of the distribution. The formula for standardization is as follows: $Z_{i}=\frac{x_{i}-\bar{x}\;}{\sigma },$ where $x_{i}$ represents each individual value of vFMI, $\bar{x}$ represents the mean value of vFMI, and σ represents the SD. This transformation yields a standardized variable with a mean of zero and an SD of one, which is suitable for subsequent statistical analyses. Missing covariate data were estimated using the multiple imputation by chained equations (MICE) method, resulting in a total of 5 imputed datasets [33,34]. Specifically, education level, income-to-poverty ratio, smoking status, and alcohol consumption were imputed, which allowed us to retain an additional 683 participants (education level: 1, income-to-poverty ratio: 373, smoking status: 2, alcohol consumption: 352). Five imputed datasets were generated and pooled according to Rubin’s rules. To assess robustness, we further conducted a sensitivity analysis comparing the results from the imputed datasets with those from complete-case analysis (CCA); the findings were consistent across both approaches (Table S1). To evaluate the plausibility of the missing at random (MAR) assumption, we compared the baseline characteristics of participants with complete covariate data (*n* = 4,847) and those with missing covariates imputed by MICE (*n* = 683) (Table S2). Additionally, we supplemented the baseline characteristics of participants with missing covariates imputed by MICE (*n* = 683) (Table S2).

Multivariable logistic regression analysis was used to estimate the relationship between vFMI *z* score and the prevalence of diabetes, DN, and CVD. The results were presented as odds ratios (ORs) with 95% confidence intervals (CIs). Model 1 was unadjusted, while model 2 was adjusted for sex, age, and race. Based on model 2, model 3 further adjusted for education level, PIR, alcohol consumption, and smoking status. Model 4 included additional adjustments for HDL-C and SBP based on model 3. Generalized additive models (GAMs) with penalized spline smoothing were first used to explore the dose–response relationships and potential nonlinearity between vFMI and the 3 outcomes. To further quantify these relationships, multivariable logistic regression models with restricted cubic splines (RCSs) were fitted using the “rcssci” package. Following package recommendations, models were specified with 4 knots and the reference probability parameter was set at 0.1. Both the original and log-transformed vFMI (natural logarithm, ln) were analyzed, and evidence of nonlinearity was formally assessed using the overall and nonlinearity *P* values. Threshold analyses were performed only when *P* nonlinear was significant, and candidate inflection points suggested by the RCS curves were further evaluated with segmented regression and bootstrap CIs. Subgroup analyses were conducted by including interaction terms between vFMI *z* score and the following factors in the logistic regression models: sex, age (≤40 years or >40 years), smoking and alcohol status, hypertension, BMI (<25.00 or ≥25.00 kg/m^2^), TG, CVD, HOMA-IR (<3.1 or ≥3.1), and HOMA-B (<100% or ≥100%).

The “mediation” package in R version 4.3.3 was used for mediation analysis to assess the mediating effects of glucose and lipid metabolism indicators (HOMA-IR, HOMA-B, and TG) on the associations between vFMI and diabetes, DN, and CVD. The analysis was adjusted for sex, age, race, education level, PIR, alcohol status, smoking status, HDL-C, and SBP.

## Results

### Baseline characteristics of participants

The baseline characteristics of the 4,847 participants, stratified by quartiles of vFMI, are presented in Table 1. The vFMI quartiles ranged from Q1 (0.07) to Q4 (0.30). Across the quartiles of vFMI, demographic and clinical characteristics varied in multiple aspects.

**Table 1.** Baseline characteristics of participants according to vFMI. Data are presented as median [Q1, Q3], mean (SD), or *n* (%).

Characteristic	Overall, *N* = 4,847	Q1 0.072 [0.009–0.100), *N* = 1,212	Q2 0.130 [0.100–0.158), *N* = 1,211	Q3 0.195 [0.158–0.235), *N* = 1,212	Q4 0.295 [0.235–0.706], *N* = 1,212	*P* value ^a^
vFMI	0.18 (0.10)	0.07 (0.02)	0.13 (0.02)	0.19 (0.02)	0.32 (0.07)	<0.001
Age	40.00 [30.00, 50.00]	29.00 [24.00, 39.00]	38.00 [29.00, 47.00]	44.00 [35.00, 52.00]	48.00 [40.00, 55.00]	<0.001
Gender, *n* (%)						<0.001
Male	2,469 (50.94)	604 (49.83)	676 (55.82)	673 (55.53)	516 (42.57)	
Female	2,378 (49.06)	608 (50.17)	535 (44.18)	539 (44.47)	696 (57.43)	
Race, *n* (%)						<0.001
Mexican American	697 (14.38)	72 (5.94)	123 (10.16)	213 (17.57)	289 (23.84)	
Other Hispanic	517 (10.67)	101 (8.33)	106 (8.75)	148 (12.21)	162 (13.37)	
Non-Hispanic White	1,747 (36.04)	447 (36.88)	386 (31.87)	434 (35.81)	480 (39.60)	
Non-Hispanic Black	1,004 (20.71)	335 (27.64)	312 (25.76)	206 (17.00)	151 (12.46)	
Other race	882 (18.20)	257 (21.20)	284 (23.45)	211 (17.41)	130 (10.73)	
Education level ^b^, *n* (%)						<0.001
Less than 9th grade	293 (6.04)	28 (2.31)	50 (4.13)	87 (7.18)	128 (10.56)	
9–11th grade	607 (12.52)	129 (10.64)	115 (9.50)	172 (14.19)	191 (15.76)	
High school graduate/GED or equivalent	1,043 (21.52)	250 (20.63)	257 (21.22)	289 (23.84)	247 (20.38)	
Some college or AA degree	1,559 (32.16)	395 (32.59)	391 (32.29)	364 (30.03)	409 (33.75)	
College graduate or above	1,345 (27.75)	410 (33.83)	398 (32.87)	300 (24.75)	237 (19.55)	
PIR ^b^, *n* (%)	2.88 (1.68)	2.88 (1.70)	3.01 (1.69)	2.85 (1.66)	2.79 (1.65)	0.072
Smoking ^b^, *n* (%)						0.006
Yes	2,010 (41.47)	455 (37.54)	473 (39.06)	525 (43.32)	557 (45.96)	
No	2,837 (58.53)	757 (62.46)	738 (60.94)	687 (56.68)	655 (54.04)	
Alcohol ^b^, *n* (%)						0.001
Yes	3,498 (72.17)	906 (74.75)	902 (74.48)	877 (72.36)	813 (67.08)	
No	1,349 (27.83)	306 (25.25)	309 (25.52)	335 (27.64)	399 (32.92)	
BMI (kg/m^2^)	28.89 (6.90)	23.15 (4.02)	27.27 (4.98)	30.08 (5.24)	35.05 (6.89)	<0.001
WC (cm)	97.45 (16.62)	81.69 (9.85)	93.65 (11.20)	101.63 (11.93)	112.81 (14.97)	<0.001
SBP (mmHg)	119.17 (15.25)	113.65 (12.87)	117.41 (14.43)	120.94 (15.01)	124.67 (16.25)	<0.001
DBP (mmHg)	71.10 (11.29)	67.10 (10.66)	70.18 (11.09)	73.32 (10.57)	73.80 (11.50)	<0.001
Glucose (mM)	5.50 [5.16, 5.88]	5.22 [4.94, 5.50]	5.38 [5.11, 5.72]	5.55 [5.22, 6.00]	5.77 [5.44, 6.44]	<0.001
Insulin (µUm/ml)	8.96 [5.73, 14.91]	5.64 [3.59, 8.02]	7.66 [5.32, 11.42]	10.36 [6.76, 15.58]	15.38 [10.33, 22.46]	<0.001
HDL cholesterol (mM)	1.29 [1.09, 1.58]	1.53 [1.27, 1.84]	1.34 [1.14, 1.63]	1.24 [1.03, 1.45]	1.16 [1.01, 1.42]	<0.001
Triglycerides (mM)	1.15 [0.79, 1.73]	0.79 [0.60, 1.08]	1.01 [0.72, 1.45]	1.33 [0.94, 2.08]	1.60 [1.17, 2.27]	<0.001
HOMA-IR	2.21 [1.35, 3.85]	1.29 [0.84, 1.89]	1.87 [1.28, 2.77]	2.62 [1.70, 4.14]	4.10 [2.67, 6.47]	<0.001
HOMA-B	89.92 [58, 145.42]	65.31 [43.75, 95.49]	78.45 [56.04, 120.33]	99.55 [64.50, 149.58]	131.53 [84.91, 185.27]	<0.001
UACR (mg/g)	5.93 [57.36, 10.00]	5.92 [4.09, 9.71]	5.20 [3.89, 8.44]	5.80 [4.12, 9.21]	7.29 [4.71, 12.31]	<0.001
eGFR (ml/min/1.73 m^2^)	103.51 (17.07)	106.47 (16.73)	104.20 (16.39)	102.19 (16.85)	101.19 (17.82)	<0.001
Hypertension, *n* (%)						<0.001
Yes	1,518 (31.31)	173 (14.27)	279 (23.03)	433 (35.72)	633 (52.22)	
No	3,329 (68.68)	1,039 (85.72)	933 (79.97)	778 (64.28)	579 (47.78)	
Diabetes, *n* (%)						<0.001
Yes	567 (11.70)	27 (2.23)	70 (5.78)	147 (12.13)	323 (26.65)	
No	4,280 (88.30)	1,185 (97.77)	1,141 (94.22)	1,065 (87.87)	889 (73.35)	
DN, *n* (%)						<0.001
Yes	136 (2.81)	9 (0.74)	9 (0.74)	34 (2.81)	84 (6.93)	
No	4,711 (97.19)	1,203 (99.26)	1,202 (99.26)	1,178 (97.19)	1,128 (93.07)	
CVD, *n* (%)						<0.001
Yes	208 (4.29)	21 (1.73)	30 (2.48)	63 (5.20)	94 (7.76)	
No	4,639 (95.71)	1,191 (98.27)	1,181 (97.52)	1,149 (94.80)	1,118 (92.24)	

**Table:** 

PIR, poverty income ratio; BMI, body mass index; SDP, systolic blood pressure; DBP, diastolic blood pressure; WC, waist circumference; UACR, urinary albumin–creatinine ratio; eGFR, estimated glomerular filtration rate; DN, diabetic nephropathy; CVD, cardiovascular disease; HDL, high-density lipoprotein; HOMA-IR, homeostatic model assessment of insulin resistance; HOMA-B, homeostatic model assessment of β-cell function

^a^ Design-based Kruskal–Wallis test; Pearson’s χ^2^: Rao & Scott adjustment.

^b^ The following variables were used MICE.

Participants in higher vFMI quartiles were generally older (Q1: 29 years versus Q4: 48 years, *P* < 0.001). Additionally, individuals in higher quartiles exhibited higher BMI, WC, SBP, DBP, fasting glucose, insulin levels, TG, HOMA-IR, and HOMA-B (all *P* < 0.001). Conversely, HDL-C levels decreased significantly across the quartiles, reaching their lowest in Q4. The racial composition showed that non-Hispanic Black and other race individuals were underrepresented in higher vFMI quartiles, whereas Mexican American participants were increasingly represented as vFMI increased (*P* < 0.001). Additionally, individuals with lower education levels (below high school) were more concentrated in the highest quartile, while the proportion of college graduates decreased significantly as vFMI increased (*P* < 0.001). Furthermore, participants in the highest quartile exhibited substantially higher prevalence rates of hypertension (52.2% versus 14.3%, *P* < 0.001), diabetes (26.6% versus 2.2%, *P* < 0.001), DN (6.9% versus 0.7%, *P* < 0.001), and CVD (7.8% versus 1.7%, *P* < 0.001) compared to those in the lowest quartile. Among the entire cohort, the prevalence of diabetes, DN, and CVD was 11.6%, 2.8%, and 4.3%, respectively.

### Associations of standardized vFMI with diabetes, DN, and CVD prevalence

The associations between vFMI and the prevalence of diabetes, DN, and CVD were assessed using multiple logistic regression models (Table 2). For diabetes, each 1-SD increase in vFMI was associated with higher risk of diabetes prevalence across all models. The fully adjusted model 4 showed an OR of 1.90 (95% CI: 1.66 to 2.17, *P* < 0.001). Quartile analysis further revealed a significantly elevated risk in Q4 compared to the lowest quartile (Q1) in model 4 (OR: 5.85, 95% CI: 3.25 to 10.55, *P* < 0.001).

**Table 2.** Multiple logistic regression analysis of the association between standardized vFMI (*z* scores, per 1-SD increase) and quartiles of vFMI with the prevalence of diabetes, diabetic nephropathy, and CVD

vFMI	Events	OR (95% CI)							
Model 1		*P* value	Model 2	*P* value	Model 3	*P* value	Model 4	*P* value
**DM**
Per-SD	4,847	2.41 (2.18–2.66)	<0.001	2.25 (1.98–2.56)	<0.001	2.20 (1.95–2.50)	<0.001	1.90 (1.66–2.17)	<0.001
Quartile									
Q1	1,212	Ref.		Ref.		Ref.		Ref.	
Q2	1,211	2.85 (1.48–5.52)	0.003	2.17 (1.11–4.22)	0.027	2.18 (1.13–4.22)	0.025	1.73 (0.91–3.29)	0.104
Q3	1,212	6.22 (3.55–10.90)	<0.001	4.17 (2.36–7.36)	<0.001	4.07 (2.33–7.11)	<0.001	2.61 (1.49–4.57)	0.002
Q4	1,212	17.93 (10.43–30.85)	<0.001	11.52 (6.43–20.66)	<0.001	10.87 (6.11–19.33)	<0.001	5.85 (3.25–10.55)	<0.001
*P* for trend		<0.001		<0.001		<0.001		<0.001	
**DN**
Per-SD	4,847	2.20 (1.85–2.60)	<0.001	2.14 (1.71–2.69)	<0.001	2.08 (1.65–2.63)	<0.001	1.78 (1.35–2.34)	<0.001
Quartile									
Q1	1,212	Ref.		Ref.		Ref.		Ref.	
Q2	1,211	0.88 (0.28–2.78)	0.832	0.64 (0.20–2.04)	0.456	0.64 (0.21–2.00)	0.449	0.51 (0.17–1.54)	0.239
Q3	1,212	4.16 (1.53–11.31)	0.007	2.66 (0.90–7.85)	0.082	2.61 (0.87–7.78)	0.092	1.66 (0.58–4.74)	0.353
Q4	1,212	9.67 (3.59–26.09)	<0.001	6.07 (1.98–18.56)	0.003	5.59 (1.80–17.35)	0.004	2.84 (0.89–9.08)	0.085
*P* for trend		<0.001		<0.001		<0.001		0.009	
**CVD**
Per-SD	4,847	1.60 (1.38–1.84)	<0.001	1.30 (1.08–1.57)	0.007	1.25 (1.03–1.53)	0.028	1.24 (0.97–1.58)	0.088
Quartile									
Q1	1,212	Ref.		Ref.		Ref.		Ref.	
Q2	1,211	1.69 (0.77–3.73)	0.196	1.22 (0.55–2.73)	0.626	1.27 (0.58–2.79)	0.555	1.25 (0.58–2.74)	0.571
Q3	1,212	4.10 (1.98–8.51)	0.001	2.46 (1.16–5.22)	0.023	2.40 (1.15–4.99)	0.023	2.36 (1.10–5.07)	0.033
Q4	1,212	5.56 (2.85–10.84)	<0.001	2.79 (1.37–5.67)	0.007	2.60 (1.26–5.35)	0.013	2.55 (1.10–5.93)	0.035
*P* for trend		<0.001		0.003		0.009		0.030	

**Table:** 

vFMI, visceral fat mass index; OR, odds ratio; CI, confidence interval; CVD, cardiovascular disease; PIR, poverty income ratio; HDL, high-density lipoprotein; SBP, systolic blood pressure

Model 1: no variables adjusted. Model 2: sex, age, and race were adjusted. Model 3: sex, age, race, education level, PIR, alcohol consumption, and smoking were adjusted. Model 4: sex, age, race, education level, PIR, alcohol consumption, smoking, HDL cholesterol, and SBP were adjusted.

Data in table: N: number of observed; % (95% CI): survey-weighted percentage (95% CI); for diabetes, diabetic nephropathy and CVD: survey-weighted OR (95% CI) *P* value.

Similarly, vFMI was strongly associated with the prevalence of DN. Each 1-SD increase in vFMI was associated with an OR of 1.78 (95% CI: 1.35 to 2.34, *P* < 0.001) in model 4. However, in the quartile analysis, the association for Q4 compared to Q1 showed an OR of 2.84 (95% CI: 0.89 to 9.08, *P* = 0.085). This indicates that the overall trend across quartiles was significant, while individual quartile comparisons were not statistically significant for DN. For CVD, vFMI demonstrated a weaker association compared to diabetes and DN. In model 1, each 1-SD increase in vFMI was associated with an OR of 1.60 (95% CI: 1.38 to 1.84, *P* < 0.001), which attenuated to 1.24 (95% CI: 0.97 to 1.58, *P* = 0.088) in model 4. Nevertheless, quartile analysis indicated that individuals in Q4 had significantly higher odds of CVD compared to Q1 in the fully adjusted model (OR: 2.55, 95% CI: 1.10 to 5.93, *P* = 0.035).

Sensitivity analyses comparing results from MICE and CCA yielded consistent findings (Table S1), supporting the robustness of the primary results.

After adjusting for BMI, the association between vFMI and diabetes, DN, and CVD was attenuated (Table S5). Specifically, the OR and *P* values of DN and CVD lost their statistical significance. Additionally, the *P* for trend of diabetes, DN, and CVD lost their statistical significance. The Pearson correlation coefficient between vFMI and BMI was 0.664 (95% CI: 0.648 to 0.679, *P* < 0.001), indicating a moderate positive linear relationship between these 2 variables. Furthermore, linear regression analysis showed that vFMI and BMI shared approximately 44% of the variance (*R*^2^ = 0.44). Despite the moderate correlation, the variance inflation factor (VIF) between vFMI and BMI was 1.78.

### The nonlinear associations between vFMI and disease

As shown in Fig.S1, the GAM curves suggested a curvilinear upward pattern for diabetes and DN, without clear U- or L-shapes, while the curve for CVD appeared approximately linear.

**Fig. 2.**
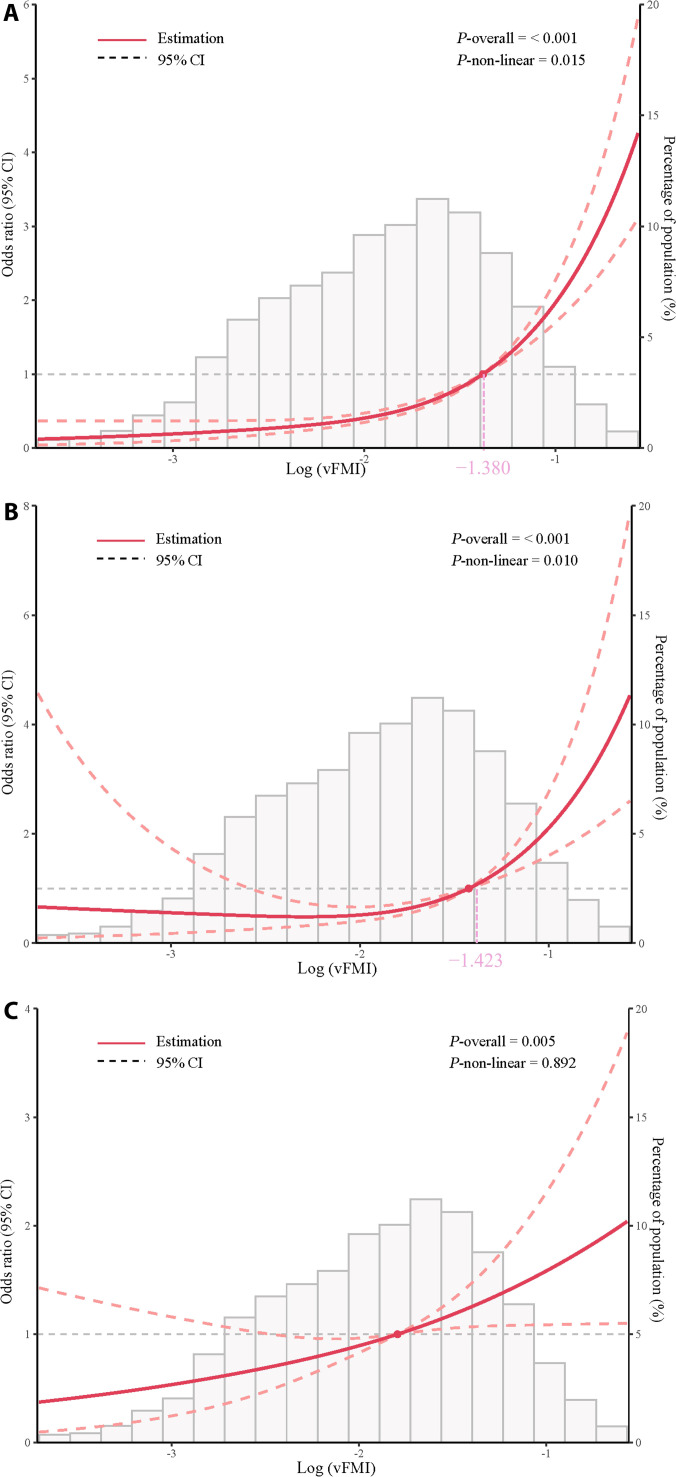
Restricted cubic spline (RCS) logistic regression of log-transformed visceral fat mass index [log(vFMI), natural logarithm] with (A) diabetes, (B) diabetic nephropathy (DN), and (C) cardiovascular disease. Red lines show odds ratios (OR) with 95% confidence intervals (CIs) (dashed). Gray bars indicate the distribution of log(vFMI). Estimated inflection points are marked for diabetes (−1.380) and DN (−1.423). *P*-overall values represent overall associations, and *P*-nonlinear values test for deviation from linearity. Adjusted for age, sex, race, education level, poverty income ratio (PIR), alcohol consumption, smoking, HDL cholesterol, and systolic blood pressure (SBP). The solid and dotted lines represent the estimated values and their corresponding 95% CIs, respectively.

To further evaluate these patterns, we fitted logistic regression models with RCS. On the original vFMI scale, the test for nonlinearity (*P* nonlinear) was significant for diabetes (*P* = 0.034) but not for DN (*P* = 0.891) or CVD (*P* = 0.272) (Fig. S2). Because vFMI was right-skewed, we also analyzed log-transformed vFMI. On this log scale, nonlinearity was significant for diabetes (*P* = 0.015) and DN (*P* = 0.010), but not for CVD (*P* = 0.892), indicating that a linear model was more appropriate for CVD (Fig. 2).

For diabetes and DN, the RCS curves showed clear thresholds. The estimated log(vFMI) thresholds were −1.380 for diabetes and −1.423 for DN, above which the odds increased more sharply. Back-transformed to the original vFMI scale, these values corresponded to 0.252 and 0.241, respectively. For CVD, although the overall association was significant (*P* overall = 0.005), the nonlinearity test was not (*P* nonlinear = 0.892). Therefore, we modeled the association as linear. In the fully adjusted logistic regression (model 4, Table 2), each 1-SD increase in standardized vFMI was associated with an OR of 1.24 (95% CI: 0.97 to 1.58, *P* = 0.088).

### Subgroup analysis

**Fig. 3.**
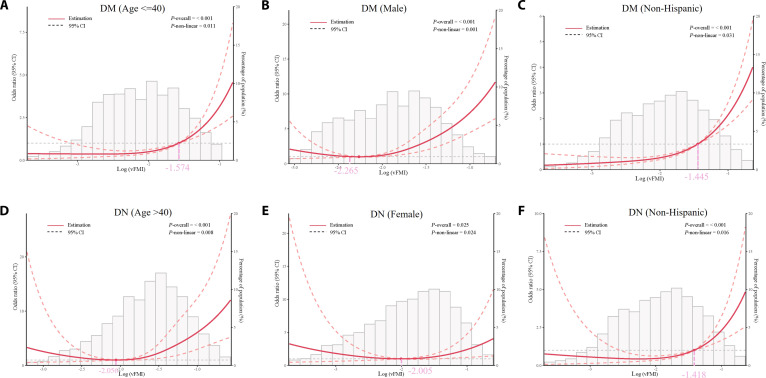
RCS logistic regression of log-transformed visceral fat mass index (vFMI) with the prevalence of diabetes (DM; A to C) and DN (D to F) in subgroup analyses. The solid red line shows the estimated OR, and the dashed line shows the 95% CI. Gray histograms indicate the distribution of log(vFMI). *P*-overall values represent overall associations, and *P*-nonlinear values test for deviation from linearity. The solid and dotted lines represent the estimated values and their corresponding 95% CIs, respectively. Adjusted for age, sex, race, education level, PIR, alcohol consumption, smoking, HDL cholesterol, and systolic blood pressure (SBP), except that sex or race was not included when stratifying by those variables.

To further investigate potential effect modifications, we conducted subgroup analyses using RCS models (Fig. 3). For diabetes, significant nonlinearity of log(vFMI) was observed among participants aged ≤40 years (*P* nonlinear = 0.011), in males (*P* nonlinear = 0.001), and in non-Hispanic individuals (*P* nonlinear = 0.031). The corresponding RCS curves identified thresholds at log(vFMI) of −1.574, −2.265, and −1.445, which back-transform to vFMI values of approximately 0.207, 0.104, and 0.236, respectively. For DN, nonlinearity was significant among participants aged >40 years (*P* nonlinear = 0.008), in females (*P* nonlinear = 0.024), and in non-Hispanic individuals (*P* nonlinear = 0.016). The thresholds were −2.056, −2.005, and −1.418 on the log scale, corresponding to vFMI values of approximately 0.128, 0.135, and 0.242. In supplementary analyses (Figs. S3 and S4), the complete set of subgroup results for diabetes and DN are presented. While some subgroups showed linear associations, the nonlinear patterns highlighted in Fig. 3 underscore that age, sex, and race/ethnicity may modify the relationship between vFMI and disease risk.

**Fig. 4.**
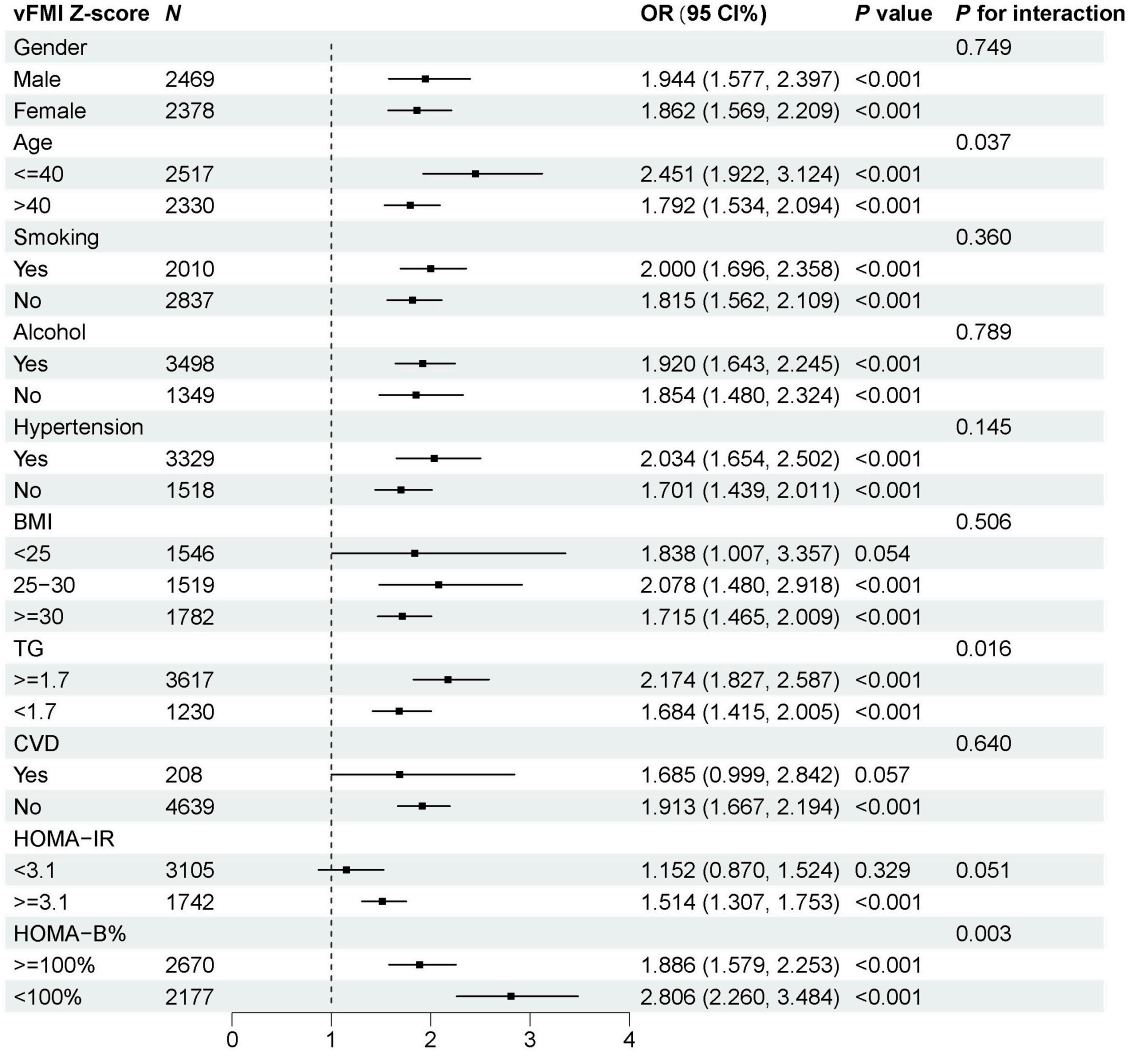
Forest plots of stratified analyses of standardized vFMI and prevalence of diabetes. Adjusted for age, sex, race, education level, PIR, alcohol consumption, smoking, HDL cholesterol, and systolic blood pressure (SBP).

Additionally, we performed subgroup analyses using interaction terms in logistic regression models (Fig. 4). Subgroup analysis demonstrated significant heterogeneity in the association between standardized vFMI *z* score and disease risk across key demographic and clinical characteristics. Notably, significant interaction effects were observed for age, TG, and HOMA-B levels. Among younger individuals (≤40 years), the association was markedly stronger (OR: 2.451, 95% CI: 1.922 to 3.124) compared to older individuals (>40 years; OR: 1.792, 95% CI: 1.534 to 2.094), with a significant interaction effect (*P* for interaction = 0.037). Similarly, in individuals with lower TG levels (<1.7 mM), vFMI exhibited a stronger relationship with significant disease risk (OR: 1.684, 95% CI: 1.415 to 2.005) than in those with higher TG levels (≥1.7 mM; OR: 2.174, 95% CI: 1.827 to 2.587) (*P* for interaction = 0.016). Furthermore, vFMI was more strongly associated with disease risk among those with higher HOMA-B levels (≥100%; OR: 1.886, 95% CI: 1.579 to 2.253) compared to those with lower levels (<100%; OR: 2.806, 95% CI: 2.260 to 3.484) (*P* for interaction = 0.003). In contrast, no significant interaction effects were identified for sex, smoking status, alcohol use, hypertension status, BMI categories, or CVD status, indicating a consistent association between vFMI *z* score and disease risk across these subgroups. These findings highlight the potential for age, TG, and β-cell function to serve as critical modifiers in the relationship between visceral adiposity and disease risk.

### Mediation analysis of the associations between vFMI and diabetes, DN, and CVD prevalence

**Fig. 5.**
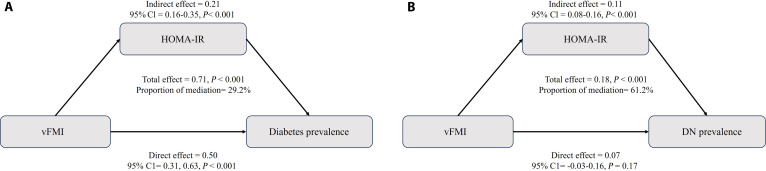
Analysis of the mediation by HOMA-IR of the associations of diabetes (A) and DN (B) prevalence. Adjusted for age, sex, race, education level, PIR, alcohol consumption, smoking, HDL cholesterol, and SBP.

For diabetes (Fig. 5A), the total effect of vFMI on DM was 0.71 (95% CI: 0.58 to 0.84, *P* < 0.001), with 29.2% of this effect mediated through HOMA-IR. The indirect effect of vFMI via HOMA-IR was 0.21 (95% CI: 0.16 to 0.35, *P* < 0.001), indicating that a significant portion of the relationship between vFMI and DM was mediated through the pathway of insulin resistance. Furthermore, the direct effect of vFMI on DM, independent of HOMA-IR, remained significant at 0.50 (95% CI: 0.31 to 0.63, *P* < 0.001), highlighting both mediated and nonmediated pathways in the association.

For DN (Fig. 5B), the total effect of vFMI was 0.18 (95% CI: 0.09 to 0.26, *P* < 0.001), with a significant 61.2% of this effect mediated through HOMA-IR. The indirect effect through HOMA-IR was 0.11 (95% CI: 0.08 to 0.16, *P* < 0.001), suggesting that insulin resistance was a predominant mediator in the relationship between vFMI and DN. In contrast, the direct effect of vFMI on DN was not statistically significant (0.07, 95% CI: −0.03 to 0.16, *P* = 0.170), indicating that HOMA-IR is accounted for most of the association.

To complement these findings, Table S4 reports mediation by additional glucose and lipid metabolism indicators (HOMA-B and TG), under both forward (vFMI→mediator→outcome) and reverse (outcome→mediator→vFMI) specifications. The analysis showed that while TG had a minor mediating role for both DM and DN, HOMA-B did not exhibit significant mediation for any of the outcomes. For CVD, the associations were primarily direct, with minimal contributions from HOMA-IR, HOMA-B, or TG. Reverse-pathway analyses yielded much smaller total effects for DM (0.047 versus 0.710 in the forward analysis) and DN (0.042 versus 0.179), although mediation proportions appeared similar.

## Discussion

This study identified significant associations between vFMI and people living with diabetes, DN, and CVD. The findings demonstrated robust correlation between high vFMI and increased risk of diabetes and its cardiovascular complications. Regression analyses revealed that after adjusting for all confounders, individuals with elevated vFMI were more likely to develop DM, DN, and CVD. These results underscore the effectiveness of vFMI as a marker for identifying high-risk individuals and suggest that reducing visceral fat could be a potential strategy to alleviate the burden of these diseases. Further analysis revealed a nonlinear relationship between vFMI and the risks of diabetes and DN. Critical vFMI thresholds, derived from log-transformed analyses, were identified: 0.252 for diabetes and 0.241 for DN. Exceeding these thresholds was associated with a sharp increase in disease risk. For CVD, the association was best described by a linear model, and no meaningful threshold was identified.

These robust findings highlight the incremental effect of visceral fat on metabolic health, reflecting potential pathways through which visceral fat accumulation exacerbates metabolic disorders via complex biological mechanisms. First, individuals with high vFMI exhibit lipolytic characteristics, releasing large amounts of free fatty acids (FFAs). These FFAs could lead to lipid accumulation, disrupt insulin signaling, and trigger metabolic disturbances [35]. Second, elevated vFMI is strongly associated with chronic low-grade inflammation. Increased proinflammatory factors such as tumor necrosis factor-α (TNF-α) and interleukin-6 (IL-6) induce systemic inflammatory responses that impair insulin signaling and damage vascular endothelium [36]. Furthermore, immune cells, particularly M1 macrophages and proinflammatory T cells, exacerbate insulin resistance and vascular inflammation by secreting proinflammatory cytokines [37]. Third, VAT promotes insulin resistance through lipolysis and reactive oxygen species (ROS) while accelerating the development of vascular complications [38].

Subgroup analyses revealed the potential modifying effects of age, TG, and HOMA-B on the associations between vFMI and the DM outcome. The association between vFMI and DM was stronger in younger individuals, suggesting a greater impact of visceral adiposity on DM risk in this age group. This is consistent with the findings of Kim and Park [39], where age-stratified analyses showed significantly higher insulin resistance ratios (IRRs) for insulin resistance in the 18 to 43 age group compared to the 44 to 65 age group. Furthermore, in individuals with TG levels ≥ 1.7 mM, the association between vFMI and adverse metabolic and cardiovascular outcomes was particularly pronounced, highlighting the compounded risk posed by elevated visceral adiposity and dyslipidemia. Evidence suggests that increased visceral fat mass is strongly correlated with elevated TG and triglyceride-glucose (TyG) index, the latter being a sensitive marker of insulin resistance. These findings underscore the role of visceral adiposity in exacerbating lipotoxicity and impairing insulin signaling, ultimately amplifying the risk of type 2 diabetes mellitus (T2DM) through mechanisms involving increased TG levels and heightened insulin resistance [40].

For individuals with HOMA-B < 100, the association between vFMI and adverse outcomes was stronger than in those with HOMA-B ≥ 100%, indicating that impaired β-cell function amplifies the detrimental effects of visceral adiposity. Reduced insulin secretion capacity exacerbates metabolic disturbances induced by excess visceral fat, as vFMI contributes to insulin resistance through proinflammatory factors like TNF-α and IL-6 while directly impairing β-cell function [41]. Conversely, individuals with HOMA-B ≥ 100% exhibit better β-cell function and compensatory insulin secretion, partially mitigating the metabolic impact of visceral fat [36]. Although no significant sex-based interaction in association between vFMI with T2DM risk was observed in this study, prior research highlights a stronger effect of visceral fat mass (VFM) on T2DM risk in women than in male, potentially due to higher insulin sensitivity, secretion capacity, and incretin response in female [42]. Those findings may reflect the higher proportion of men in intermediate vFMI groups (Q2 and Q3), potentially masking sex-specific differences seen in more extreme cases.

The mediation analysis in this study revealed that HOMA-IR plays a significant mediating role in the associations of vFMI with DM and DN, but contributed minimally for CVD. Although reverse-pathway analyses produced mediation proportions of similar magnitude, these were based on much smaller total effects compared with the forward analyses, suggesting limited explanatory value. This contrast supports the plausibility that the observed associations are more likely to follow the forward pathways (vFMI→HOMA-IR→disease) rather than reverse causality. Notably, vFMI is a key driver of HOMA-IR. A previous study highlighted that visceral fat significantly increases HOMA-IR by promoting hepatic lipid accumulation and chronic low-grade inflammation, thereby exacerbating metabolic disturbances [43]. Elevated HOMA-IR has been consistently associated with DM and its complications in several studies. A previous study found that increased HOMA-IR significantly raises the risk of T2DM, with individuals in the highest quintile having a 2.8-fold higher risk compared to those in the lowest quintile [44]. Regarding DN, a research demonstrated that prolonged elevations in HOMA-IR were closely linked to renal function deterioration, with insulin resistance significantly increasing the risk of DN [45]. Furthermore, HOMA-IR was identified as a critical risk factor for CVD, where each unit increase in HOMA-IR substantially elevated the risk of CVD (OR: 1.56) [46].

In recent years, research on the relationship between VFM and diabetes has increased significantly. Some studies have utilized DXA to assess visceral fat levels and explore their associations with diabetes, insulin resistance, and metabolic syndrome. These studies consistently found a significant positive correlation between visceral fat accumulation and diabetes. A study demonstrated that high visceral fat levels significantly increase the risk of diabetes, with assessments made using both direct measures of visceral fat and spinal DXA to quantify abdominal fat [47]. Another study highlighted that visceral fat is a stronger predictor of diabetes risk than BMI, particularly in men [48]. Furthermore, current research in Asian populations has confirmed a significant association between vFMI and diabetes risk, especially among individuals with higher visceral fat levels [17].

The study has several strengths. First, this study utilized standardized vFMI to eliminate the influence of body size differences. This approach enables a more accurate comparison of metabolic health risks among individuals with varying body types. Second, this study utilized NHANES 2011–2018 data, which includes approximately 35,000 multi-ethnic U.S. adults, providing broad racial and geographic representativeness. Third, this study employed RCS to investigate the nonlinear relationship between VFM and diabetes risk, identifying threshold effects of diabetes risk associated with increasing VFM. Fourth, this study provides evidence supporting the mediating role of HOMA-IR in the relationship between VFM and diabetes, DN, and CVD. Finally, this study highlighted the heightened susceptibility of younger populations to the effects of visceral fat.

The study has several limitations. First, despite adjusting for multiple confounding factors, unmeasured or residual confounding cannot be completely excluded. Variables that might influence vFMI and associated outcomes, such as dietary patterns, physical activity levels, and the use of specific medications like fibrates, statins, and antidiabetic drugs, warrant further investigations. These unmeasured factors may have contributed to the observed associations. Second, although our analytic cohort included 4,847 participants with valid VAT data, a large number of participants were excluded due to missing VAT information. To evaluate potential selection bias, we compared the analytic cohort with 13,611 excluded individuals who had available anthropometric data (Table S3). Excluded participants had lower mean height, weight, BMI, and WC compared with included participants. This pattern reflects the heterogeneous reasons for DXA exclusion in NHANES, which include both extreme obesity (equipment limits, obesity-related image noise) and younger or smaller participants (e.g., adolescents and incomplete scans). Thus, the excluded group does not represent a uniformly more obese population but rather a heterogeneous set of individuals for whom DXA scanning could not be completed. This limitation suggests that our analytic cohort may underrepresent both extremes of body size. Third, we did not include BMI in our primary models because our exposure, vFMI, is already height-standardized and highly correlated with BMI. Including both would raise concerns of multicollinearity and potential over-adjustment of body size-related pathways, complicating interpretation of the overall effect of visceral adiposity. However, we recognize that the attenuation of associations after adjusting for BMI in our sensitivity analyses is likely due to the independent contributions of both vFMI and BMI to the outcomes, rather than multicollinearity. Both vFMI and BMI should be carefully considered as independent predictors in future analyses. Our hierarchical models therefore focused on demographic, socioeconomic/lifestyle, and clinical risk factors, with HDL-C and SBP considered as partially mediated adjustments. Future studies may explore alternative modeling strategies (e.g., unscaled VAT mass with BMI adjustment or residual-based approaches) to disentangle the effects of visceral versus general adiposity. Fourth, while NHANES provides a representative sample of the U.S. population, the generalizability of these findings to other populations with different ethnic, cultural, and lifestyle characteristics may be limited. Fifth, insulin resistance is a complex physiological condition that can be assessed using multiple indices, such as HOMA-IR, fasting insulin levels, and the glucose–insulin ratio, each capturing different aspects of insulin sensitivity and secretion. In this study, we employed HOMA-IR as a mediator in the analysis. However, incorporating other indices of insulin resistance into future mediation models may provide a more nuanced understanding of the relationship between vFMI and metabolic complications. Lastly, as this study utilized cross-sectional data from the NHANES database, it is important to note that causal relationships cannot be established. The inability to capture disease duration accurately due to the nature of the dataset limits the exploration of temporal associations between vFMI and disease onset. Future longitudinal studies that track disease progression and consider disease duration are necessary to confirm the temporal relationships between visceral fat accumulation and the development of metabolic and vascular complications.

## Conclusion

This study demonstrates that vFMI is significantly associated with DM, DN, and CVD. These findings highlight the potential of vFMI as a valuable anthropometric indicator for metabolic and vascular complications. Additionally, vFMI provides a precise assessment of visceral adiposity, overcoming limitations inherent in traditional metrics such as BMI or WC. Furthermore, the identification of thresholds and the mediating role of HOMA-IR contribute to an understanding of the mechanisms. The distinct age-related sensitivity observed in younger populations underscores the necessity for targeted screening and intervention strategies in this group. These findings not only reinforce the role of visceral adiposity as a crucial risk factor but also suggest avenues for future research and clinical applications focusing on early prevention and personalized management of metabolic and vascular diseases.

## Ethical Approval

NHANES has been approved by the National Center for Health Statistics Ethics Review Board, and all participants were provided informed written consent at enrollment.

## Data Availability

No datasets were generated or analyzed during the current study.
