# Supplementary material for: Association of Visceral Fat Mass Index with Diabetes and Vascular Complications: NHANES 2011–2018 Cross-Sectional Study
Source: Health Data Sci. 2026 Mar 10;6:0441. doi: 10.34133/hds.0441 (PMC12972505; doi:10.34133/hds.0441)
Supplement: Supplementary 1 — Figs. S1 to S4 Tables S1 to S5 [file hds.0441.f1.docx]

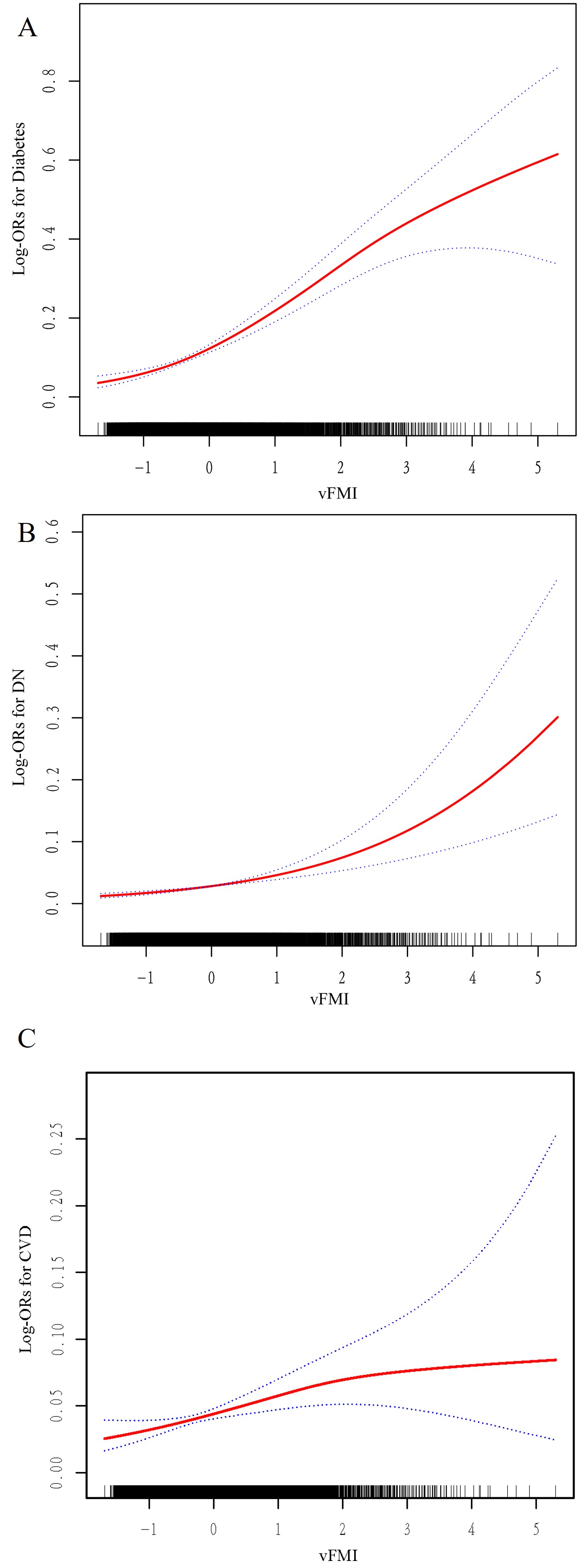


**Figure S1.** **Association between standardized vFMI and the prevalence of diabetes (A), diabetic nephropathy (B), and cardiovascular disease (C) based on GAM models and smooth curve fitting (penalized spline method).**

Adjusted for age, sex, race, education level, poverty income ratio (PIR), alcohol consumption, smoking, HDL cholesterol, and systolic blood pressure (SBP). The solid and dotted lines represent the estimated values and their corresponding 95% CIs, respectively.


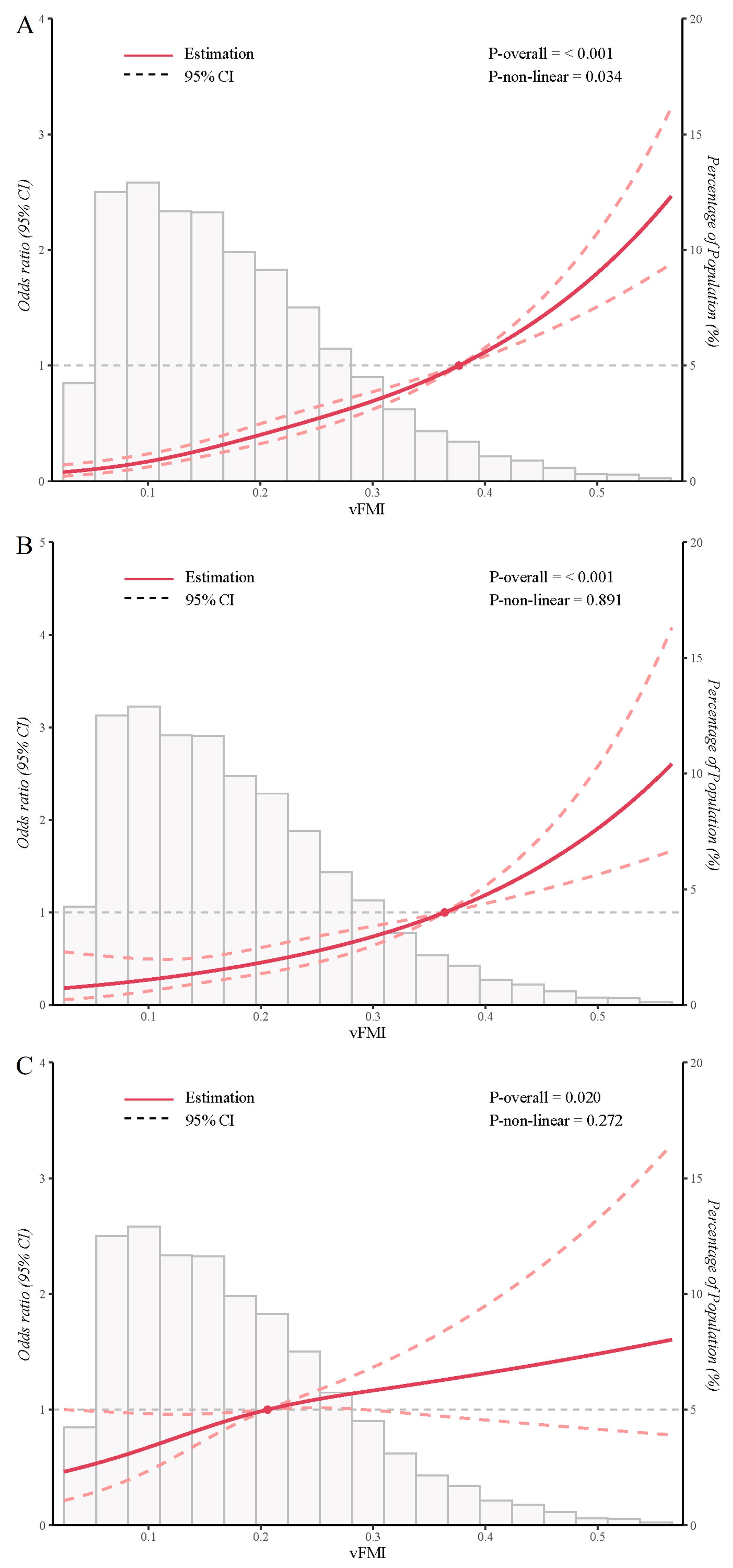


**Figure S2. Restricted cubic spline (RCS) logistic regression of visceral fat mass index (vFMI) with diabetes (A), diabetic nephropathy (B), and cardiovascular disease (C).**

Red lines show odds ratios with 95% CI (dashed); gray bars indicate the distribution of vFMI. P-overall and P-nonlinear values are shown in each panel.

Abbreviation: vFMI, visceral fat mass index.

Adjusted for age, sex, race, education level, poverty income ratio (PIR), alcohol consumption, smoking, HDL cholesterol, and systolic blood pressure (SBP). The solid and dotted lines represent the estimated values and their corresponding 95% CIs, respectively.


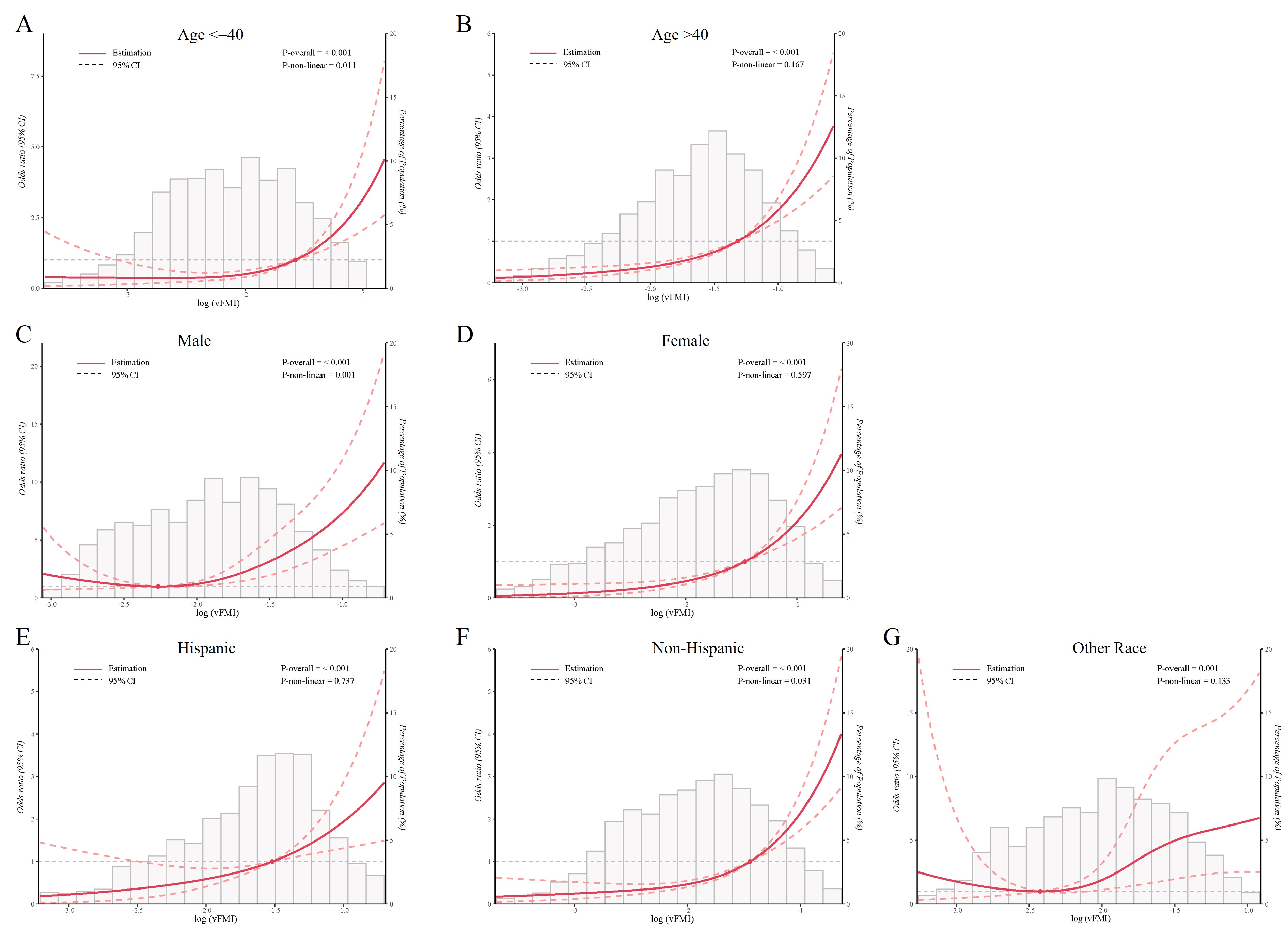


**Figure S3. Restricted cubic spline (RCS) logistic regression analyses of log-transformed visceral fat mass index (vFMI) with the prevalence of diabetes (DM) across subgroups.**

Panels show results stratified by age (A: ≤40 years; B: >40 years), sex (C: male; D: female), and race/ethnicity (E: Hispanic; F: non-Hispanic; G: other). The solid red line represents the estimated odds ratio (OR), the dashed line shows the 95% confidence interval (CI), and the gray histogram indicates the distribution of log(vFMI). P-overall and P-nonlinear values are reported in each panel.

Abbreviation: vFMI, visceral fat mass index.

Adjusted for age, sex, race, education level, poverty income ratio (PIR), alcohol consumption, smoking, HDL cholesterol, and systolic blood pressure (SBP), except that sex or race was not included when stratifying by those variables. The solid and dotted lines represent the estimated values and their corresponding 95% CIs, respectively.


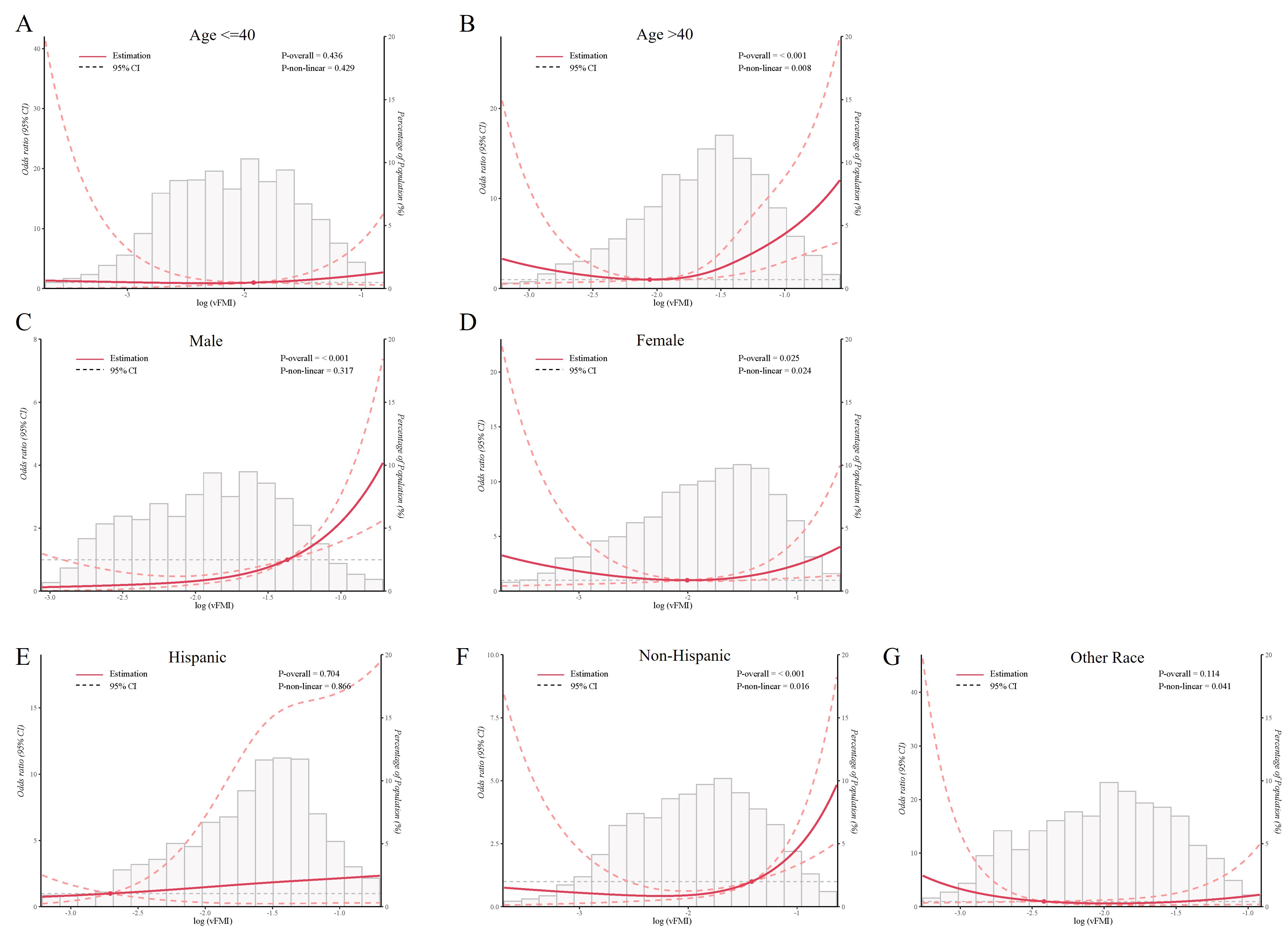


**Figure S4. Restricted cubic spline (RCS) logistic regression analyses of log-transformed visceral fat mass index (vFMI) with the prevalence of diabetic nephropathy (DN) across subgroups.**

Panels show results stratified by age (A: ≤40 years; B: >40 years), sex (C: male; D: female), and race/ethnicity (E: Hispanic; F: non-Hispanic; G: other). The solid red line represents the estimated odds ratio (OR), the dashed line shows the 95% confidence interval (CI), and the gray histogram indicates the distribution of log(vFMI). P-overall and P-nonlinear values are reported in each panel.

Abbreviation: vFMI, visceral fat mass index.

Adjusted for age, sex, race, education level, poverty income ratio (PIR), alcohol consumption, smoking, HDL cholesterol, and systolic blood pressure (SBP), except that sex or race was not included when stratifying by those variables. The solid and dotted lines represent the estimated values and their corresponding 95% CIs, respectively.

**Table S1.** Sensitivity analysis comparing results from multiple imputation (MICE) and complete-case analysis (CCA).

| vFMI Z-score | Model 4 (MICE) | | | | Model 4 (CCA) | | | |
| --- | --- | --- | --- | --- | --- | --- | --- | --- |
|  | N | | OR (95% CI) | P-value | N | | OR (95% CI) | P-value |
| DM |  |  | |  |  |  | |  |
| Per-SD | 4847 | 1.90 (1.66, 2.17) | | <0.001 | 4164 | 1.93 (1.66, 2.25) | | <0.001 |
| Quartile |  |  | |  |  |  | |  |
| Q1 | 1212 | Ref. | |  | 1041 | Ref. | |  |
| Q2 | 1211 | 1.73 (0.91, 3.29) | | 0.104 | 1041 | 1.99 (1.06, 3.72) | | 0.038 |
| Q3 | 1212 | 2.61 (1.49, 4.57) | | 0.002 | 1041 | 2.96 (1.72, 5.08) | | <0.001 |
| Q4 | 1212 | 5.85 (3.25, 10.55) | | <0.001 | 1041 | 6.56 (3.64, 11.80) | | <0.001 |
| P for trend |  | <0.001 | |  |  | <0.001 | |  |
| DN |  |  | |  |  |  | |  |
| Per-SD | 4847 | 1.78 (1.35, 2.34) | | <0.001 | 4164 | 1.77 (1.32, 2.38) | | <0.001 |
| Quartile |  |  | |  |  |  | |  |
| Q1 | 1212 | Ref. | |  | 1041 | Ref. | |  |
| Q2 | 1211 | 0.51 (0.17, 1.54) | | 0.239 | 1041 | 0.47 (0.14, 1.62) | | 0.2406 |
| Q3 | 1212 | 1.66 (0.58, 4.74) | | 0.353 | 1041 | 1.67 (0.49, 5.72) | | 0.4206 |
| Q4 | 1212 | 2.84 (0.89, 9.08) | | 0.085 | 1041 | 2.64 (0.71, 9.79) | | 0.0202 |
| P for trend |  | 0.009 | |  |  | 0.020 | |  |
| CVD |  |  | |  |  |  | |  |
| Per-SD | 4847 | 1.24 (0.97, 1.58) | | 0.088 | 4164 | 1.26 (0.99, 1.60) | | 0.065 |
| Quartile |  |  | |  |  |  | |  |
| Q1 | 1212 | Ref. | |  | 1041 | Ref. | |  |
| Q2 | 1211 | 1.25 (0.58, 2.74) | | 0.571 | 1041 | 1.33 (0.58, 3.06) | | 0.504 |
| Q3 | 1212 | 2.36 (1.10, 5.07) | | 0.033 | 1041 | 2.34 (1.01, 5.41) | | 0.054 |
| Q4 | 1212 | 2.55 (1.10, 5.93) | | 0.035 | 1041 | 2.64 (1.05, 6.64) | | 0.045 |
| P for trend |  | 0.030 | |  |  | 0.037 | |  |

Model 4: sex, age, race, education level, PIR, alcohol consumption, and smoking, HDL cholesterol, SBP were adjusted.

Data in table: N: Number of observed; For Diabetes, Diabetic Nephropathy and CVD: survey-weighted OR (95%CI) p-value.

Abbreviations: vFMI Visceral fat mass index, OR odds ratio, CI Confidence interval, CVD Cardiovascular disease, PIR Poverty income ratio, HDL High-Density lipoprotein, SBP Systolic blood pressure, MICE Multiple imputation by chained equations; CCA Complete-case analysis.

**Table S2.** Baseline Characteristics of Participants with Missing Covariate Data Imputed by MICE.

| Characteristic | Q1 0.077 (0.018 –0.101)  N = 171 | Q2 0.127 (0.102 –0.156)  N = 171 | Q3 0.193 (0.157 –0.230)  N = 170 | Q4 0.287 (0.231–0.706)  N = 171 | *P* value^1^ |
| --- | --- | --- | --- | --- | --- |
| vFMI | 0.07 (0.02) | 0.13 (0.02) | 0.19 (0.02) | 0.31 (0.07) | <0.001 |
| Age, | 31.00 [25.00, 40.00] | 37.00 [30.50, 46.50] | 42.00 [34.25, 51.00] | 48.00 [40.00, 54.50] | <0.001 |
| Gender, n (%) |  |  |  |  | 0.034 |
| Male | 82 (47.95) | 91 (53.22) | 74 (43.53) | 65 (38.01) |  |
| Female | 89 (52.05) | 80 (46.78) | 96 (56.47) | 106 (61.99) |  |
| Race, n (%) |  |  |  |  | <0.001 |
| Mexican American | 8 (4.68) | 17 (9.94) | 34 (20.00) | 55 (32.16) |  |
| Other Hispanic | 17 (9.94) | 25 (14.62) | 28 (16.47) | 26 (15.20) |  |
| Non-Hispanic White | 52 (30.41) | 26 (15.20) | 31 (18.24) | 34 (19.88) |  |
| Non-Hispanic Black | 53 (30.99) | 42 (24.56) | 29 (17.06) | 28 (16.37) |  |
| Other Race | 41 (23.98) | 61 (35.67) | 48 (28.24) | 28 (16.37) |  |
| BMI (kg/m^2^) | 23.10 (4.33) | 26.94 (5.11) | 29.47 (4.49) | 34.13 (6.76) | <0.001 |
| WC (cm) | 81.57 (10.25) | 92.50 (11.29) | 98.86 (10.78) | 109.81 (15.03) | <0.001 |
| SBP (mm Hg) | 113.51 (13.84) | 115.47 (12.21) | 120.61 (13.36) | 124.61 (18.70) | <0.001 |
| DBP (mm Hg) | 66.54 (10.65) | 69.61 (11.07) | 74.12 (10.25) | 73.36 (10.49) | <0.001 |
| Glucose(mmol/L) | 5.16 [4.94, 5.50] | 5.42 [5.11, 5.77] | 5.55 [5.22, 6.00] | 5.88 [5.47, 6.55] | <0.001 |
| Insulin (uUm/ml) | 6.24[3.83, 8.98) | 8.08[5.53, 11.59] | 10.07[7.02, 16.20] | 13.72 [9.61, 21.98] | <0.001 |
| HDL Cholesterol (mmol/L) | 1.60 [1.27, 1.89] | 1.29 [1.11, 1.58] | 1.27[1.09, 1.47] | 1.19 [1.01, 1.40] | <0.001 |
| Triglycerides (mmol/L) | 0.81 [0.58, 1.08] | 0.97 [0.68, 1.40] | 1.23[0.92, 1.77] | 1.42 [1.04, 2.18] | <0.001 |
| HOMA-IR | 1.42 [0.84, 2.10] | 1.92 [1.34, 2.85] | 2.53 [1.73, 3.98] | 3.78 [2.64, 6.44] | <0.001 |
| HOMA-B | 73.92 [46.89, 108.87] | 84.07 [57.15, 132.15] | 98.62 [65.48, 157.65] | 114.38 [69.49, 186.59] | <0.001 |
| UACR (mg/g) | 6.25 [3.98, 10.43] | 5.36 [3.85, 8.38] | 6.55 [4.31, 10.65] | 7.58 [5.14, 12.96] | 0.402 |
| eGFR (mL/min/1.73 m²) | 107.74 [96.19, 119.63] | 106.89 [93.69, 117.50] | 105.56[92.03, 115.97] | 106.09 [96.38, 112.96] | <0.001 |
| Hypertension, n (%) |  |  |  |  | <0.001 |
| Yes | 31 (18.10) | 31 (18.10) | 55 (32.40) | 85 49.7) |  |
| No | 140 (81.90) | 140 (81.90) | 778 (67.60) | 579 (50.3) |  |
| Diabetes, n (%) |  |  |  |  | <0.001 |
| Yes | 4 (2.34) | 6 (3.51) | 18 (10.60) | 41 (24.00) |  |
| No | 167 (97.66) | 165 (96.49) | 152 (89.40) | 130 (76.00) |  |
| DN, n (%) |  |  |  |  | <0.001 |
| Yes | 1 (0.58) | 1 (0.58) | 2 (1.18) | 11 (6.43) |  |
| No | 170 (99.42) | 170 (99.42) | 168 (98.82) | 160 (93.57) |  |
| CVD, n (%) |  |  |  |  | <0.001 |
| Yes | 4 (2.34) | 3 (1.75) | 5 (2.94) | 18 (10.53) |  |
| No | 167 (97.66) | 168 (98.25) | 165 (97.06) | 153 (89.47) |  |

Data are presented as Median [Q1, Q3], mean (SD) or n (%), ^1^Design-based Kruskal-Wallis test; Pearson's X^2: Rao & Scott adjustment

Abbreviations: BMI Body mass index, SDP Systolic blood pressure, DBP Diastolic blood pressure, WC Waist circumference, UACR urinary albumin-creatinine ratio, eGFR Estimated glomerular filtration rate, DN Diabetic Nephropathy, CVD cardiovascular disease.

**Table S3.** Comparison of baseline characteristics between included participants with valid VAT data and excluded participants without VAT data in NHANES (n=18,458).

| **Characteristic** | **Overall**  N = 18458^1^ | **Included (VAT available)**  N = 4847^1^ | **Excluded (VAT missing)**  N = 13611^1^ | **p-value**^2^ |
| --- | --- | --- | --- | --- |
| Gender |  |  |  | <0.001 |
| Male | 8888 (48%) | 2469 (51%) | 6419 (47%) |  |
| Female | 9570 (52%) | 2378 (49%) | 7192 (53%) |  |
| Age | 41 ± 27 | 40 ± 12 | 41 ± 30 | <0.001 |
| Race Ethnicity |  |  |  | <0.001 |
| Mexican American | 2789 (15%) | 697 (14%) | 2092 (15%) |  |
| Other Hispanic | 2004 (11%) | 517 (11%) | 1487 (11%) |  |
| Non-Hispanic White | 6434 (35%) | 1747 (36%) | 4687 (34%) |  |
| Non-Hispanic Black | 4325 (23%) | 1004 (21%) | 3321 (24%) |  |
| Other Race | 2906 (16%) | 882 (18%) | 2024 (15%) |  |
| BMI | 26 ± 8 | 29 ± 7 | 25 ± 8 | <0.001 |
| Waist Circumference | 88 ± 25 | 97 ± 17 | 85 ± 26 | <0.001 |
| Height | 152 ± 27 | 168 ± 10 | 146 ± 28 | <0.001 |
| Weight | 65 ± 32 | 82 ± 22 | 59 ± 33 | <0.001 |
| ^1^n (%); Mean ± SD | | | | |
| ^2^Pearson's Chi-squared test; Welch Two Sample t-test | | | | |

**Table S4.** Mediation Analysis of Glucose and Lipid Metabolism Indicators in the Associations of vFMI with DM, DN, and CVD Prevalence: Comparison of Forward and Reverse Effects.

|  |  |  | | **Mediation effect (95% CI), P value** | |  |  |  | |  | |  |
| --- | --- | --- | --- | --- | --- | --- | --- | --- | --- | --- | --- | --- |
|  | Indicator | Total effect (Forward) | Total Effect (Reverse) | Indirect effect (Forward) | Indirect effect (Reverse) | | Direct effect (Forward) | | Direct effect (Reverse) | | Mediation (Forward) | Mediation (Reverse) |
| **DM prevalence** |  |  |  |  |  | |  | |  | |  |  |
|  | HOMA-IR | 0.710 (0.576,0.840) <0.001 | 0.047 (0.038, 0.060) <0.001 | 0.207 (0.155, 0.350) <0.001 | 0.012 (0.008, 0.020) <0.001 | | 0.503 (0.314, 0.630) <0.001 | | 0.035 (0.022, 0.040) <0.001 | | 29.20% | 25.70% |
|  | HOMA-B | 0.710 (0.576,0.840) <0.001 | 0.047 (0.038, 0.060) <0.001 | -0.001(-0.105,0.000) <0.001 | -0.001(-0.001,0.000) <0.001 | | 0.711 (0.586, 0.880) <0.001 | | 0.047 (0.039, 0.060) <0.001 | | NA^C^ | NA^C^ |
|  | TG | 0.710 (0.576,0.840) <0.001 | 0.047 (0.038, 0.060) <0.001 | 0.030 (0.012, 0.050) <0.001 | 0.002 (0.001, 0.000) <0.001 | | 0.680 (0.542, 0.810) <0.001 | | 0.045 (0.035, 0.050) <0.001 | | 4.20% | 4.80% |
| **DN prevalence** |  |  |  |  |  | |  | |  | |  |  |
|  | HOMA-IR | 0.179 (0.091,0.260) <0.001 | 0.042 (0.022, 0.060) <0.001 | 0.110 (0.079, 0.160) <0.001 | 0.025 (0.018, 0.040) <0.001 | | 0.069 (-0.030, 0.160) 0.170 | | 0.017 (-0.007, 0.040) 0.170 | | 61.20% | 60.00% |
|  | HOMA-B | 0.179 (0.091,0.260) <0.001 | 0.042 (0.022, 0.060) <0.001 | 0.000 (-0.026, 0.020) 0.950 | 0.000 (-0.004, 0.001) 0.590 | | 0.179 (0.089, 0.270) <0.001 | | 0.042 (0.021, 0.060) <0.001 | | 0% | 0% |
|  | TG | 0.179 (0.091,0.260) <0.001 | 0.042 (0.022, 0.060) <0.001 | 0.016 (0.005, 0.040) 0.002 | 0.004 (0.002, 0.010) <0.001 | | 0.163 (0.071,0.250) <0.001 | | 0.038 (0.017, 0.060) <0.001 | | 8.70% | 9.60% |
| **CVD prevalence** |  |  |  |  |  | |  | |  | |  |  |
|  | HOMA-IR | 0.106 (0.022, 0.200) 0.014 | 0.016 (0.003, 0.030) 0.014 | 0.004 (-0.010, 0.030) 0.689 | 0.001 (-0.001, 0.010) 0.330 | | 0.102 (0.015, 0.200) 0.024 | | 0.015 (0.002, 0.030) 0.024 | | 3.40% | 7.30% |
|  | HOMA-B | 0.106 (0.022, 0.200) 0.014 | 0.016 (0.003, 0.030) 0.014 | 0.000 (-0.004, 0.020) 0.653 | 0.000 (-0.001, 0.000) 0.280 | | 0.106 (0.018, 0.200) 0.017 | | 0.016 (0.002, 0.003) 0.017 | | 0% | 0% |
|  | TG | 0.106 (0.022, 0.200) 0.014 | 0.016 (0.003, 0.030) 0.014 | 0.002 (-0.006, 0.020) 0.721 | 0.001 (-0.001, 0.000) 0.490 | | 0.104 (0.017, 0.200) 0.018 | | 0.015 (0.003, 0.030) 0.018 | | 1.60% | 3% |

NA^c^: Not applicable due to null or unstable estimates.

Adjust for: sex, age, race, education level, PIR, alcohol status, smoking status, HDL cholesterol, SBP.

Abbreviation: TG triglycerides, DM diabetes, DN diabetic nephropathy, CVD cardiovascular disease, HOMA-IR homeostatic model assessment indices for insulin resistance, HOMA-B% homeostatic model assessment indices fo β-cell function.

**Table S5**. Logistic Regression Analysis of vFMI and Cardiometabolic Outcomes with Additional Adjustment for BMI.

| vFMI | Events | OR (95%CI) |  |
| --- | --- | --- | --- |
|  |  | Model 5 | P-value |
| **DM** |  |  |  |
| Per-SD | 4847 | 1.37(1.21, 1.56) | <0.001 |
| Quartile |  |  |  |
| Q1 | 1212 | Ref. |  |
| Q2 | 1211 | 1.28(0.81, 2.08) | 0.303 |
| Q3 | 1212 | 1.65(1.06, 2.64) | 0.032 |
| Q4 | 1212 | 2.36(1.48, 3.86) | <0.001 |
| P for trend |  | 0.632 |  |
| **DN** |  |  |  |
| Per-SD | 4847 | 1.27 (1.03, 1.56) | 0.027 |
| Quartile |  |  |  |
| Q1 | 1212 | Ref. |  |
| Q2 | 1211 | 0.43 (0.16, 1.14) | 0.085 |
| Q3 | 1212 | 0.91 (0.42, 2.14) | 0.821 |
| Q4 | 1212 | 1.16 (0.52, 2.82) | 0.733 |
| P for trend |  | 0.264 |  |
| **CVD** |  |  |  |
| Per-SD | 4847 | 1.01 (0.84, 1.22) | 0.906 |
| Quartile |  |  |  |
| Q1 | 1212 | Ref. |  |
| Q2 | 1211 | 0.89 (0.50, 1.61) | 0.196 |
| Q3 | 1212 | 1.20 (0.69, 2.14) | 0.525 |
| Q4 | 1212 | 1.09 (0.59, 2.06) | 0.792 |
| P for trend |  | 0.123 |  |

Model 5: sex, age, race, education level, PIR, alcohol consumption, and smoking, HDL cholesterol, SBP, BMI were adjusted.

Data in table: N: Number of observed; % (95%CI): survey-weighted percentage (95% CI); For Diabetes, Diabetic Nephropathy and CVD: survey-weighted OR (95%CI) p-value.

Abbreviations: vFMI Visceral fat mass index, OR odds ratio, CI Confidence interval, CVD Cardiovascular disease, PIR Poverty income ratio, HDL High-Density lipoprotein, SBP Systolic blood pressure, BMI Body mass index.
